# Editorial: Organs integrative endocrinology - the interplay between the pathways regulating endocrine organs

**DOI:** 10.3389/fendo.2023.1323185

**Published:** 2023-11-28

**Authors:** Bidesh Mahata, Rifka Schulman-Rosenbaum, Fawzy A. Saad

**Affiliations:** ^1^ Department of Pathology, University of Cambridge, Cambridge, United Kingdom; ^2^ Division of Endocrinology, Department of Medicine, Long Island Jewish Medical Center, Donald and Barbara Zucker School of Medicine at Hofstra/Northwell, New Hyde Park, NY, United States; ^3^ Department of Drug Discovery, Saad Pharmaceuticals, Tallinn, Estonia

**Keywords:** integrative endocrinology, skin & adipose endocrine, muscle & bone endocrine, brain endocrine, pancreas endocrine, ovary & testes endocrine, parathyroid endocrine

Since the birth of modern endocrinology in the late nineteenth century, which can be flagged to 1891 (1), advances in the field have led to steady disappearance of the frontiers of traditional endocrinology. In the views of modern endocrinology, hormones secreted from endocrine organs or tissues or cells seem to work in an integrative manner. Brain, adrenal, skin, muscle, bone, gonads, pancreas, adipose, liver, thyroid, parathyroid, immune and other tissue hormones have feedback loops and networks to regulate the physiology. In fact, most physiological and psychological tasks initiate the interplay between organs. As expected, in the clinic and health care system an integrative psychosomatic approach toward endocrine problems is under consideration. No doubt, the future of endocrinology is moving toward an integrative and systems level understanding of interconnected endocrine pathways. To increase the holistic understanding of the molecular pathways regulating endocrine organs and tissues we invited relevant papers to run this Research Topic. We are thankful to all the authors who have contributed.

This topic showcases three literature reviews and five original research articles. Constitutively they provide topical and valuable new insight into integrative bone-, adipose-, ovary-, pancreas-, thyroid-, and brain- endocrine pathways which are vital for health problems such as osteoporosis, diabetes, chronic kidney disease, obesity, thyroid disease, and skin lesions/manifestations.

In the mini review on endocrine regulation of bone health, Chang et al. summarized the mechanisms of how physical exercise impacts on osteoporosis and provides new proposals for the prevention and treatment of osteoporosis. Bone development, its homeostasis and pathologies such as osteoporosis, are dynamic processes. Due to a lack of spatio-temporal resolution at the single-cell level, the bone dysfunction aetiologies caused by diseases such as normal aging, osteoporosis, and the metabolic bone disease associated with chronic kidney disease remain incompletely understood. By combining genomic, transcriptomic, and predictive metabolic profiling approaches, Agoro et al. demonstrated how cortical bone transcriptomics define novel osteolineage gene sets altered in chronic kidney disease. They identified osteolineage genes associated with distinct cell populations of osteoblast precursors, mature osteoblasts and osteocytes and demonstrated how these three cortical bone cell populations were dysregulated in a mouse model of chronic kidney disease prior to the development of cortical porosity. In another bone endocrine article, Liang et al. studied the relationship between the serum irisin level and bone microarchitecture. Irisin is a newly discovered hormone that plays a role in bone-muscle crosstalk (2). The authors found that postmenopausal women with lower serum irisin levels have a higher fall risk, weaker muscle strength, and higher cortical porosity. Moreover, serum irisin level has a positive association with cortical volumetric bone mineral density, but it is affected by factors such as age. In type 1 diabetes, risk factors associated with impaired bone health contribute to increased risk of fracture. Sochett et al. studied the impairment of bone health in young adults with type 1 diabetes. Their findings imply that a combined approach of vitamin D supplement, treatment of secondary hyperparathyroidism, along with metabolic control may reduce incident fractures.

Thyroid is considered one of the key endocrine regulators for skin homeostasis. Multiple skin diseases are associated with thyroid hormone dysregulation. Hypothyroidism, hyperthyroidism, and thyroid cancer can have an array of cutaneous manifestations as revealed by numerous studies in recent years, particularly in the last decade. Cohen et al. summarise the updates in any new skin disease findings and treatments between 2010 and 2022, and discussed the new directions that remain to be unravelled.

Recently much effort in endocrinology research have been given to obesity and type 2 diabetes, partly regulated by integrated endocrine interaction of pancreas, adipose and neural (brain) tissue. Sarcopenia, an age-related progressive loss of muscle mass and strength, and obesity are two highly prevalent conditions in older adults. To investigate the association between body fat (BF%) and sarcopenia in older adults with type 2 diabetes mellitus (T2DM) and potential link with increased levels of inflammatory indicators and insulin resistance, Sun et al. conducted a cross-sectional study with 543 subjects. They concluded that the higher body fat (BF%) was linked to an increased risk of sarcopenia in older adults with T2DM, suggesting the importance of assessing BF% rather than BMI alone to manage sarcopenia. In another study Huang et al. found that the visceral fat (VF) correlates with insulin secretion and sensitivity in Chinese patients with T2DM and worsens insulin sensitivity independent of body mass index (BMI) and subcutaneous fat. The study raises the concern that practitioners should not undermine the risk of insulin resistance and β-cell dysfunction in their patients entirely based on BMI but consider fat distribution as well. The intervention of obesity is multifaceted and diverse. Application of acupuncture is one of the mostly debated approach for obesity. Landgraaf et al. reviewed acupuncture as multi-targeted therapy for obesity. This narrative review outlines the evidence for this neuro-endocrine-immune interplay in the pathophysiology of obesity. Furthermore, the existing experimental and clinical evidence of acupuncture as a multi-targeted therapy for obesity is explained and future research perspectives are discussed.

Altogether, this topic evidenced a dense interplay between the pathways regulating endocrine organs such as brain, pancreas, skin, and bone (summarised diagrammatically in the **Figure 1**). In the future, increasing integrated approaches in analytical investigations are anticipated to visualise a holistic interaction map of endocrine pathways in tissue homeostasis and dysfunction.

**Figure 1:**
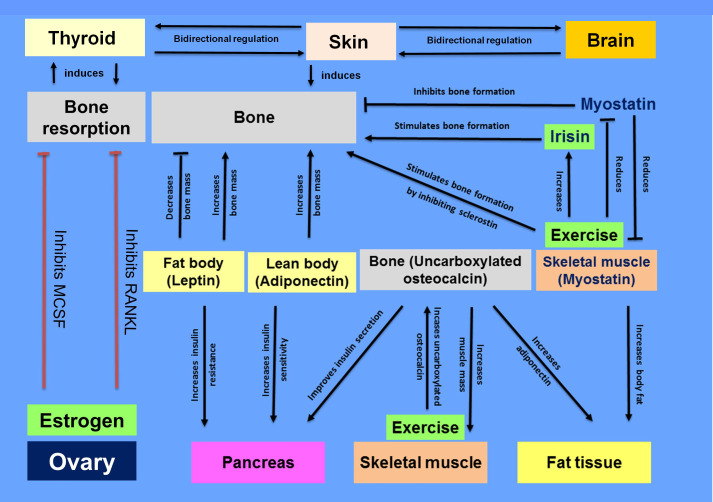
Interplay between the pathways regulating endocrine organs such as brain, pancreas, skin, adipose, ovary and bone.

## Author contributions

BM: Writing – original draft, Visualization. RS-R: Writing – review & editing. FS: Visualization, Writing – review & editing.
